# Utilization of long acting and permanent contraceptive methods and associated factor among women of reproductive age in west Guji zone, Southwest Ethiopia

**DOI:** 10.1186/s12978-022-01337-6

**Published:** 2022-01-31

**Authors:** Eden Dagnachew Zeleke, Dawit Getachew Assefa, Tigist Tekle Woldesenbet, Rediet Gido, Nebiyu Mengistu, Wondwosen Molla

**Affiliations:** 1 Department of Midwifery, Institute of Health, Bule Hora University, Bule Hora, P.O.BOX 22495/1000, Ethiopia; 2 Department of Nursing, College of Health Science, Dilla University, Dilla, Ethiopia; 3 Department of Public Health, College of Health Science, Hawassa University, Hawassa, Ethiopia; 4 Department of Midwifery, College of Health Science, Dilla University, Dilla, Ethiopia; 5 Department of Psychiatry, College of Health Science, Dilla University, Dilla, Ethiopia

**Keywords:** Utilization, Family planning, Contraception, Long acting and permanent contraceptive methods

## Abstract

**Background:**

Increasing access to family planning helps to ensure the reproductive right, decrease unintended pregnancy, improve the health and nutritional status of children, reduction of maternal mortality, and enhance longer birth spacing. However, there is continually low utilization of long acting and permanent contraceptive methods among low and middle-income countries. This study aimed to assess the utilization of long acting and permanent contraceptive methods (LAPMs) and associated factors among women of reproductive age in the West Guji Zone, Ethiopia.

**Methods:**

An institution-based cross-sectional study was carried out among 507 women of reproductive age in the West Guji Zone, Southwest Ethiopia from April 15 to May 15, 2018. Data were collected by a structured, pretested, and interview-based questionnaire with open ended and closed ended questionnaire, then entered, and analyzed by SPSS Version 20. Bivariable and multivariate logistic regression analyses were carried out. A 95% confidence interval (CI) AND P-value < 0.05 was considered to declare statistically significant variables.

**Result:**

The current utilization of LAPMs at West Guji zone among the reproductive-aged group was found to be 51.1%. More than the median of participants had negative altitude (72.4%) and poor knowledge (57%) towards the LAPMs. Educational status of women, the number of alive children, acceptance of utilization of LAPMs, how treated by other staff, and waiting time during service delivery are significant determinant factors of LAPMs.

**Conclusion:**

Overall, more than half of women had a negative attitude and poor knowledge of LAPMs. Educational status of women, the number of alive children, acceptance of utilization of LAPMs, how treated by other staff, and waiting time during service delivery were factors affecting utilization of LAPMs. Therefore, sustained, and appropriate information on LAPMs should be provided to raise knowledge and build the attitude of women and the community. Treating the clients with respect, reducing the waiting time, and collaborative work with health extension worker will enhance utilization of LAPMs.

## Plain language summary

Low utilization of LAPMs has been continually reported in low- and middle-income countries. Unintended pregnancy and unsafe abortion might be related with unmet need of contraceptive.

This study aimed to assess level of utilization of LAPMs among reproductive aged women in West Guji Zone Ethiopia. The findings from this research will help different stake holder by providing the level of current utilization of LAPMs and help them on planning how to strength the utilization by refereeing the associated factors.

507 Respondents were selected systematically: of which 51.1% of them utilize one of LAPMs currently.

In conclusion the utilization may be affected by educational status of women, number of alive children, acceptance LAPMs by women, how treated by other staff during service delivery, and waiting time to get the service.

## Background

Family planning allow people to exercise their basic human right to freely decide when to have children, improve families nutrition outcome by limiting their number of children, promote their health and wellbeing, inhibit the distribution of sexually transmitted disease like HIV/AIDS, support women’s and girl’s education and empowerment and contribute to economic development of the country [1].

Reproductive age women have been facing enormous challenges related to unmet need of contraceptive which might be because of lack of awareness to protect their health, unavailability of service, lack their decision-making role on post ponding childbearing. These obstacles might abort their future promising opportunities and it vast from complications during pregnancy and birth because of unintended pregnancy to lower their education accomplishment which affects their future plan and lead to facing risk for their child [2]. Africa records the highest proportion (45%) of unintended pregnancy next to Latin America (74%) [2]. Although about half of unintended pregnancies in Latin America and 46% in Africa results in subsequent unsafe abortion [3]. In Ethiopia the unmet need of contraceptive is 15.1% and only 62.5% (median) demand for family planning was satisfied with modern methods [4]. Large proportion of unintended pregnancy also revealed in urban and rural areas of Ethiopia, i.e. 34% in Debre Markos [5], Arsi Negele Woreda 41.5% [6] and 23.5% in Debre Birhan [7], 36.4% Addis Ababa [8], and also 29% in sub-Saharan Africa [9]. According to different studies unintended pregnancy might related with not using contraceptive [10], their knowledge regarding intrauterine device [8] and women autonomy on deciding to use contraceptive [5, 6]. Unintended pregnancy could be caused by failure to use modern contraceptive methods.

According to WHO 2019 report, global 270 million adolescent have unmet need for contraceptive [11]. Greater unmet need of contraceptive showed in sub-Saharan countries compared to the globe. A recent study also shown that there is continually low utilization of long acting and permanent contraceptive method in low- and middle-income countries [10].

To ensure the reproductive right, decrease unintended pregnancy, improve health and nutritional status of children, reduction of maternal mortality, and enhance longer birth spacing, increasing access to family planning plays a significant role [4] and also decreases the need for unsafe abortion [12]. Furthermore, it has promising effect on ensuring long time protection without need of any further responsibility by the user once applied. However, even though the modern contraceptive use was improved in Ethiopia, the utilization of long acting and permanent family planning services is still low because of numerous factors. Thus, there is a need to assess utilization of LAPMs and associated factor among women of reproductive age in West Guji Zone. The finding of this study will be useful for governmental and non-governmental organizations to take intervention measures and set appropriate plans to improve the existing utilization of LAPMs and health managers at a higher level and health professionals to understand the extent of the utilization level of LAPMs. The findings will also enhance the capacity of planning and decision making to look for possible solutions to solve the problem in collaboration with concerned stakeholders to provide future appropriate LAPMs services in the district as well as in the west Guji Zone.

## Objectives

### General objective

- To assess utilization of long acting and permanent contraceptive methods and associated factor among women of reproductive age in west Guji Zone, 2018.

### Specific objectives

- To determine levels of utilization of long acting and permanent family planning methods.
- To identify factors associated with utilization of long acting and permanent family planning methods.

## Method

### Study area

The study was conducted in governmental health institutions which are found in Oromia west Guji zone. In west Guji zone there are three hospitals in which two of them were functional (Bule Hora General Hospital & Kercha primary hospital) & one is not (Melka Soda Hospital) at the time of study. There were 44 health centers in the zone, of those 3 were nonfunctional.

### Study period

The study was conducted from April 15 to May 15, 2018.

### Study design

Hospital based cross sectional design was employed.

### Inclusion and exclusion criteria

#### Inclusion criteria

- All women of reproductive age come to the selected health facilities to get Family planning service.
- Women whose age from 15 to 49.

#### Exclusion criteria

- Women who were not willing to answer.
- Women who were not come to get family planning service.

### Variables

#### Dependent variable

- Utilization of long acting and permanent family planning methods.

#### Independent variable

- Age
- Education
- Access to service
- Parity
- Husband opinion
- Satisfaction with information given
- Method accepted
- Knowledge and
- Attitude towards LAPMS of method

### Population

*Source population* All women come to health institution.

*Study population* All women come to get family planning service in selected health institution.

### Sample size determination and sampling procedure

#### Sample size quantitative data

A single population proportion formula was used to determine the prevalence of LAPMs by taking proportion of long acting and permanent contraceptive methods in Mekele town, Tigray region, Ethiopia (P = 12.3%) [13]. Other inputs considered was 95% confidence level, margin of error of 3% and 10% non-response rate: giving a final sample size of 507.

#### Sampling technique/procedures

West Guji zone health facilities were divided in to two (the hospitals & the health centers). One hospital & 21 health centers were selected randomly.

### Data collection instruments

A structured and pretested interview-based questionnaire with open ended and closed ended questions was used to collect the data.

### Data processing and analysis

Data was entered cleaned, coded, and analyzed using SPSS versions 20. Both descriptive and analytical statistical procedures were utilized. Bivariable and multivariate logistic regression analyses were carried out. P-value < 0.05 was considered to declare statistically significant variables.

### Quality control

The principal investigator was given introduction for the data collectors and supervised all activities for the collected data completeness and clarity if there was any problem; it was addressed on the next day.

### Operational definitions

Very good knowledge: those who scored 80% and above distinct features of LAPMs from knowledge questions.

Good knowledge: those who scored 60 up to 79% distinct features of LAPMs from knowledge questions.

Poor knowledge: those who scored less than 60% features of any of the LAPMs from knowledge questions.

Positive attitude: those who scores above mean to the correct answers from attitude measuring LAPMs questions.

Negative attitude: those who score mean and below mean to the correct answers from attitude measuring LAPMs questions.

Acceptance of utilization of LAPMs: respondents’ willingness to use LAPMS.

## Results

A total of 507 reproductive age women were interviewed from 21 health centers and 1 hospital that found in west Guji zone with 100% response rate. Majority of the respondents’ 283 (55.8%) were between the age of 25 and 34 years, Table 1.

**Table 1:** Socio-demographic characteristics of respondent (N = 507)

Variable	Frequency(number)	Percent
Age of the women		
15–24	182	35.9
25–34	283	55.8
35–49	42	8.3
Religion		
Orthodox	142	28
Muslim	66	13
Protestant	294	58
Catholic	5	1
Educational level of women		
Informal	47	9.3
Grade 1–8	131	25.8
Grade 9–12	143	28.2
College and university	40	7.9
Illiterate	146	28.8
Martial status		
Married	462	91.1
Single	24	4.7
Divorced	7	1.4
Widowed	8	1.6
Separated	6	1.2
Occupation		
Government employee	189	37.3
NGO employee	2	0.4
Private business (merchant)	72	14.2
Housewife	162	32.0
Student	40	7.9
Daily laborer	32	6.3
Farmer	10	2
Monthly income (ETB)		
< 499	84	16.6
500–1499	192	37.9
1500–2499	111	21.9
2500–3499	84	16.6
3500–4499	14	2.8
> 4500	22	4.3

*ETB* Ethiopian Birr

### Obstetric characteristics of the respondents in west Guji zone

Majority of the respondents 404 (79.7%) were found to have married between the age of 14–24 and at their first birth 374 (73.8%) of the respondents were 14–24 years old with mean ± SD of age 22 ± (3.65) years old, Table 2.

**Table 2:** Obstetric characteristics of the respondents in west Guji zone (N = 507)

Variables	Frequency(number)	Percent
Age at time of marriage		
14–24	404	79.7
25–35	79	15.6
Yet not married	25	4.7
Age at time of first birth		
14–24	374	73.8
25–35	81	16
Yet not give birth	52	10.3
Gravidity (number of pregnancy)		
1	108	21.3
2–5	262	51.7
6 and above	87	17.2
No pregnancy	50	9.9
Parity (no of birth)		
1–2	160	31.6
3 and above	295	58.2
No children	52	10.3
Future desire of more babies		
1–2	33	6.5
3–4	152	30
5–6	125	24.7
> 6	93	18.3

### Utilization of long acting and permanent methods in west Guji zone

During the study period 259 (51.1%) of the respondent were using LAPMs. Most of the respondent 229 (45.2%) were currently using Implant, followed by IUCD 25(4.9%), Table 3.

**Table 3:** Utilization of long acting and permanent methods in west Guji zone (N = 507)

LAPMs	Frequency	Percentage
Are you currently using LAPMs		
Yes	259	51.1
Implant	229	45.2
IUCD	25	4.9
Tubal ligation	5	1
Vasectomy	0	0
No	248	48.9

*IUCD* Intrauterine contraceptive device, *LAPMS* long acting and permanent contraceptive method

### Knowledge of the respondent on long acting and permanent method

Three hundred thirty-seven (66.5%) of women know at least one method of modern family planning method. Injectable followed by implant was the most known method and male sterilization (vasectomy) is the least known among the modern family planning which was 323(63.7%), 265(52.3%), and 48(9.5%) respectively. Two hundred eighty (55.2%) of women know at least one LAPMs among which implant was the most known, Table 4.

**Table 4:** Knowledge of the respondent on long acting and permanent method in west Guji zone (N = 507)

Do you know any modern family planning methods?	Frequency	Percent
Yes	337	66.5
Pill as modern family planning	197	38.9
IUCD as modern family planning	132	26
Injectable as modern family planning	323	63.7
Tubal ligation as modern family planning	58	11.4
Vasectomy as modern family planning	48	9.5
Implant as modern family planning	265	52.3
Lactation as modern family planning	52	10.3
Condom as modern family planning	133	26.2
Do you know about LAPMs?	280	55.2
IUCD as long acting & permanent method	126	24.9
Implant as long acting and permanent method	277	54.6
Tubal ligation as long acting and permanent method	59	11.6
Vasectomy as long acting and permanent method	45	8.9

*IUCD -* Intrauterine contraceptive device, *LAPMS* - long acting and permanent contraceptive method

Furthermore, majority of the respondents (43%) were believed that long acting and permanent contraceptive method have used for child spacing and 147 (29%) have good knowledge on long acting and permanent contraceptive. However, 71 (14%) of them had a very good knowledge and 289 (57%) of them had poor knowledge respectively, Table 5.

**Table 5:** Knowledge of the respondent on LAPMs in west Guji zone

	Yes	No	Don’t know
Does IUCD can prevent pregnancies for more than 10 years	215(42.4%)	150(29.6%)	142(28%)
Do you think IUCD appropriate for female at high risk of getting STI	128(25.2%)	223(44%)	156(30.8%)
IUCD don’t interference with sexual intercourse or desire?	190(37.5%)	166(32.7%)	151(28.9%)
Do you think IUCD is immediately reversible? (become pregnant quickly when removed)	196(38.7%)	156(30.8%)	155(30.6%)
IUCD does not can cause cancer?	212(41.8%)	142(28%)	153(30.2%)
Do you think Implant can prevent pregnancies for 3–5 years?	276(54.4%)	80(15.8%)	151(29.8%)
Do Implants require minor surgical procedure during insertion and removal?	265(52.3%)	90(17.8%)	152(30.0%)
Do you think Implants is immediately reversible?	242(47.7%)	101(19.9%)	164(32.3%)
Does female sterilization need an operation to be performed?	215(42.4%)	117(23.1%)	175(34.5%)
LAPMs do not cause ectopic pregnancy?	201(39.6%)	123(24.3%)	183(36.1%)

*IUCD* intrauterine contraceptive device, *LAPMS* long acting and permanent contraceptive method, *STI* sexually transmitted infection

### Altitude of the respondent toward LAPMS

Three hundred eighty-one (75.1%) of the respondent accept long acting and permanent family planning methods and 276 (54.4%) were supported by their husband. However, three hundred thirty-seven (72.4%) of the respondent had poor altitude toward long acting and permanent family planning methods and one hundred forty (27.6%) of the respondent have good altitude, Table 6.

**Table 6:** Attitude of respondents on utilization of LAPMs in west Guji zone, 2018 G.C

	Disagree	Not sure	Agree
The insertion and removal of implant is highly painful	187 (36.9%)	131 (25.8)	189 (37.3%)
Insertion of IUCD causes loss of pregnancy	122 (24.1%)	167 (32.9%)	218 (43%)
Using IUCD prevents from doing heavy work	170 (33.5%)	165 (32.5%)	172 (33.9%)
Operation for female sterilization is dangerous	128 (25.2%)	203 (40%)	176 (34.7%)
IUCD and implant cause infertility	196 (38.7%)	186 (36.7)	125 (24.7%)
The IUCD will get stuck in my uterus	183 (36.1%)	179 (35.3%)	145 (28.6%)
Nulliparous women shouldn’t have used an IUCD	138 (27.2%)	172 (33.9%)	197 (38.9%)
IUCD will not fit in my uterus	196 (38.7%)	191 (37.7%)	120 (23.7%)

*IUCD* Intrauterine contraceptive device

### Practice of long acting and permanent methods

Utilization of long acting and permanent contraceptive method summarized on Table 7.

**Table 7:** Practice of respondents on long acting and permanent contraceptive methods of west Guji Zone (N = 507)

Practice of respondents	Frequency	Percent
Have you ever used LAPMs?		
Yes	217	42.8
No	290	57.2
If yes, Have you ever used implant?	205	40.4
If yes, Have you ever used IUCD?	12	2.4
If yes, Have you ever used vasectomy?	0	0
If yes, Have you ever used tubal ligation?	0	0
Reason for stop using LAPMs		
Fear of side effect	109	21.5
Partners disapprove	111	21.9
Medical problem	20	3.9
Shift to other method	34	6.7
I want a child	25	4.9
Religious opposition	22	4.3
Don’t know	43	8.5
Reason for currently using long acting and permanent contraceptive method		
Have enough child	59	11.6
Pressure from my husband	37	7.3
Want to space	123	24.3
Advice from health professional	144	28.4
Reason for not practicing LAPMS		
Fear of side effect	113	22.3
Service unavailability	9	1.8
Partner disapprove	147	29
Medical problem	32	6.3
Shift to other method	40	7.9
Lack of knowledge	79	15.6

*IUCD -* intrauterine contraceptive device, *LAPMS -* long acting and permanent contraceptive method

One hundred seventy-nine (35.3%) of the respondent were get the service from health center which is followed by sixty-four (12.6%) were get from hospital. The rest were get the service from NGO clinic four (0.8%) and health post twelve (2.4%).

### Perceived service quality of respondents on long acting and permanent contraceptive methods

Participant’s response on service quality summarized on Table 8.

**Table 8:** Perceived service quality of respondents on long acting and permanent contraceptive methods of West Guji zone in 2018 (N = 507)

		Frequency	Percent
During your visit to health facility how were you treated by the other staff?	Very well	236	46.5
	well	110	21.7
	Not very well/poorly	159	31.4
	There was no other staff	2	0.4
How long did you wait to get service at the facility?	< 15 min	14	2.8
	16–30 min	129	25.4
	31–45 min	122	24.1
	46–60 min	67	13.2
	61–90 min	66	13
	91–120 min	66	13
	> 120 min	38	7.5
	Don’t know	5	1
How do you feel about your waiting time?	No waiting time	73	14.4
	Reasonable/short time	218	43
	Too long	209	41.2
	Don’t know	7	1.4
Did the provider ask if you were having a problem with the method that you used?	Yes	460	90.7
	No	47	9.3
Have you had a problem with your method?, that you wanted to discuss with your provider	Yes	421	83
	No	86	17
Did the provider try to understand the nature of your problem?	Yes	332	65.5
	No	175	48.3
Did the provider suggest what you should do (action you should take) to resolve the problem?	Yes	302	59.6
	No	205	40.2
Were you satisfied with the advice or treatment that you received for your problem?	Yes	443	87.4
	No	64	12.6

### Factors associated with utilization of LAPMs among women of reproductive age group in west Guji zone, 2018

In the binary logistic regression analysis family size, educational level of women, parity, accept long acting and permanent contraceptive methods, ever use LAPMs, husband opinion, how treated by other staff, service satisfaction, reasonable waiting time, and level of knowledge were statistically associated with utilization of LAPMs.

After controlling the effect of other variables (confounders), the likelihood of a utilization of LAPMs was 88.3% less likely for women who have attended high school than women who have attended informal education. Besides, the likelihood of a utilization of LAPMs was 91.8% less likely for women’s who had more than three children than women who had less than three children. However, the likelihood of a utilization of LAPMs was 97.1% less likely for women’s who haven’t accepted LAPMs than women who accepted. Consistently, the likelihood of a utilization of LAPMs was 26% less likely for women were not treated well by service provider than women who were treated well. Additionally, the likelihood of a utilization of LAPMs was 78% and 90.2% less likely for women who have waited to get service for reasonable and long time, respectively than women who got the service on arrival, Table 9.

**Table 9:** Factors associated with utilization of LAPMs among women of reproductive age group in west Guji zone, 2018

Variables	Utilization of LAPM		COR(95% CI)	AOR(95% CI)
	Yes	No		
Family size				
1–3	210	225	1	1
≥ 4	49	23	0.438(0.258–0.744)	1.54(0.21–11.34)
Educational level of women				
Informal	21	26	1	
Grade 1–8	70	61	0.704(0.36–1.375)	0.78(0.172–3.53)
Grade 9–12	115	28	0.197(0.097–0.399)	0.116 (0.023–0.593) *
College or university	30	10	0.269(0.12–0.674)	0.446(0.055–3.644)
Parity				
1–2	45	115	1	1
≥ 3	192	103	0.210(0.138–0.319)	0.082(0.026–0.253)*
Accept LAPMs				
Yes	239	142	1	1
No	20	106	0.112(0.067–0.189)	0.029(0.005–0.162)*
Husband opinion				
Support	164	112	1	1
Oppose	56	90	0.425(0.28–0.64)	0.74(0.179–3.1)
How treated by other staff				
Well	234	112	1	1
Not very well	25	136	0.088(0.054–0.143)	0.06(0.017–0.213)*
Reasonable waiting time				
No waiting time	42	31	1	1
Reasonable/short	182	36	0.038(0.023–0.064)	0.022(0.005–0.087)*
Too long	35	181	0.143(0.079–0.257)	0.098(0.026–0.376)*
Service satisfaction				
Yes	222	187	0.511(0.325–0.803)	1.1(0.28–4)
No	37	61	1	1
Ever use LAPM				
Yes	130	87	0.536(0.375–0.766)	0.94(0.33–2.68)
No	129	161	1	
Level of knowledge				
Poor knowledge	115	174	1	1
Good knowledge	41	30	0.484(0.286–0.819)	1.04(0.21–5.2)
Very good knowledge	103	44	0.282(0.185–0.432)	0.5(0.166–1.58)

*AOD* adjusted odds ratio, *CI* confidence interval, *COR* crude odds ratio, *LAPMS* long acting and permanent contraceptive method, *Significant *P* value < 0.05

## Discussion

The finding of these study indicate that educational status women, number of alive children (parity), accept utilization of LAPMs, how treated by other staff during service delivery and waiting time for service are significant determinant factor of long acting and permanent family planning method.

In this study the overall the current use of LAMP at west Guji zone was 51.1%. This is very high when compared to research done in Bombe district, Gonder City, Dendi district and Janamora District which were 16.3%, 34.7%, 17.6% and 12.9% respectively [14–17] and which is less than the study done in Ilu Aba Bor Zone, Aksum city, and Lay-Armachiho district, Amhara regional state, Ethiopia that was 62.2%, 52.1%, and 65.4% respectively [18–20]. This difference could be in study setting, time of the study, the deployments of health extension worker which strengthen the awareness of LAPMs at community level and the government attention towards these methods increases the utilization [21].

Another factor affecting utilization of long acting and permanent family planning method is educational level of women. Women who join high school (Grade 9–12) 88.3% (AOR 0.116(0.023–0.593)) less likely to use long acting and permanent contraceptive method than women with informal education. This is result contradict with the result from a study done at Bombe district women who have educational level primary (1–8)/above AOR 3.7 95% CI (1.7–7.9) more likely to use LAPMs than women had no formal education, Gonder city, and Lay-Armachiho district, Amhara regional state, Ethiopia [14, 17, 19].

In this study 337 (65.5%) of women know at least one method of modern family planning method which is very low when compared to the study done at Debre Berhan Town which is (459) 92% of the respondents were able to mention at least one method of modern family planning method [22]. The least known method as LAPMs is vasectomy (8.9%) and implant were very known method under LAPMs (54.6%) which is followed by IUCD 24.9% and tubal ligation (11.6%), the result was consistent with a result reported from a study conducted in Indonesia, which was positioned closest to the attributes ‘easy to use’ and ‘easy to get’ [23]. These is very low when compared to the study done at Gesuba town in which vasectomy is least known (16.6%) and Implant is most known which is followed by IUCD and tubal ligation which is 69.3%, 47.9% and 18.8% respectively [24].

In this study women who didn’t accept utilization of long acting and permanent methods 97.1% less likely to use LAPMs AOR 0.029 95% CI (0.005–0.162) than women who accept. women with more than three live children 91.8% less likely to use LAPMs than those who had 1–2 live children with AOR 0.082 95% CI (0.026–0.253) which was consistent with a study done in a rural kebeles of Nunu Kumba District [25]. But, contradicted with a study done at Debre Berhan District, women with 3 and above and 1 child is 2 times more likely to use LAPMs than who had no children with AOR 2.41(0.14–41.43 and AOR 2.74 95% CI (0.22–34.62), respectively [26].

Regarding quality of service, women who were treated not very well with other staff 94% less likely use long-acting permanent contraceptive method than those treated very well with AOR 0.06 95% CI (0.017–0.213). Women who believes there were no waiting time and reasonable waiting time for getting the service 0.098 and 0.022 time use long acting and permanent contraceptive method than women who believed waiting time was too long with AOR 0.098 95% CI (0.026–0.376) and AOR 0.022 95% CI (0.005–0.087). Regarding the altitude of participants towards long acting and permanent contraceptive method, our finding was higher than finding from Debre Berhan District in which 52.5% had negative altitude [26].

## Conclusion

Overall, more than half of women had a negative attitude and poor knowledge of LAPMs. Educational status of women, the number of alive children, acceptance of utilization of LAPMs, how treated by other staff, and waiting time during service delivery were factors affecting utilization of LAPMs. Therefore, sustained, and appropriate information on LAPMs should be provided to raise knowledge and build the attitude of women and the community. Treating the clients with respect, reducing the waiting time, and collaborative work with health extension worker will enhance utilization of LAPMs.

## Data Availability

Data will be available upon request from the correspondence authors.

## Abbreviations

AOR: Adjusted odds ratio
COR: Crudes odds ratio
FGD: Focused Group Discussion
IUCD: Intrauterine contraceptive device
LAPMs: Long acting and permanent methods
SD: Standard deviation
STI: Sexually transmitted infection
WHO: World Health Organization
